# Knockdown of Alpha-1 Antitrypsin with antisense oligonucleotide does not exacerbate smoke induced lung injury

**DOI:** 10.1371/journal.pone.0246040

**Published:** 2021-02-04

**Authors:** Kyle Stearns, Monica Goldklang, Rui Xiao, Tina Zelonina, Keith Blomenkamp, Jeffery Teckman, Jeanine M. D’Armiento

**Affiliations:** 1 College of Physicians and Surgeons, Columbia University Medical Center, New York, New York, United States of America; 2 Department of Biochemistry and Molecular Biology, Saint Louis University. St. Louis, Missouri, United States of America; National Yang-Ming University, TAIWAN

## Abstract

Alpha-1 Antitrypsin (AAT) is a serum protease inhibitor that regulates increased lung protease production induced by cigarette smoking. Mutations in the *Serpina1* gene cause AAT to form hepatoxic polymers, which can lead to reduced availability for the protein’s primary function and severe liver disease. An AAT antisense oligonucleotide (ASO) was previously identified to be beneficial for the AATD liver disease by blocking the mutated AAT transcripts. Here we hypothesized that knockdown of AAT aggravates murine lung injury during smoke exposure and acute exacerbations of chronic obstructive pulmonary disease (COPD). C57BL/6J mice were randomly divided into 4 groups each for the smoking and smoke-flu injury models. The ASO and control (No-ASO) were injected subcutaneously starting with smoking or four days prior to influenza infection and then injected weekly at 50 mg/kg body weight. ASO treatment during a 3-month smoke exposure significantly decreased the serum and lung AAT expression, resulting in increased *Cela1* expression and elastase activity. However, despite the decrease in AAT, neither the inflammatory cell counts in the bronchoalveolar lavage fluid (BALF) nor the lung structural changes were significantly worsened by ASO treatment. We observed significant differences in inflammation and emphysema due to smoke exposure, but did not observe an ASO treatment effect. Similarly, with the smoke-flu model, differences were only observed between smoke-flu and room air controls, but not as a result of ASO treatment. Off-target effects or compensatory mechanisms may account for this finding. Alternatively, the reduction of AAT with ASO treatment, while sufficient to protect from liver injury, may not be robust enough to lead to lung injury. The results also suggest that previously described AAT ASO treatment for AAT mutation related liver disease may attenuate hepatic injury without being detrimental to the lungs. These potential mechanisms need to be further investigated in order to fully understand the impact of AAT inhibition on protease-antiprotease imbalance in the murine smoke exposure model.

## Introduction

Alpha-1 antitrypsin (AAT) is a serum protease inhibitor that targets the proteases produced during injury that are ultimately responsible for structural destruction in the lung. The protease-antiprotease paradigm has long been recognized and earlier research has identified neutrophil elastase as one of the most potent proteases that mediates destruction in the lung [1, 2]. Alpha-1 antitrypsin inhibits neutrophil elastase activity [3] and prevents the degradation of elastin [4], which helps to maintain the integrity of the extra-cellular matrix in the lung. Imbalances between tissue damaging proteases and their inhibitors such as AAT leads to lung destruction and the development of chronic obstructive pulmonary disease (COPD) [5].

AAT deficiency (AATD) was the first identified genetic predisposition to COPD [6]. Broad screening studies suggest 2% of patients with COPD have AATD [7]. AATD carriers (Pi*MZ) can account for as high as 17.8% among patient populations [8, 9]. Moreover, the disease has several clinical manifestations; COPD that is attributed to a loss of anti-protease activity, and cirrhosis that is attributed to accumulation of misfolded Z-AAT protein within the hepatocyte [10].

Unlike the single gene *Serpina1* that encodes AAT in humans, there are five genes (*Serpina1a~f*) that encode AAT in mice. Although a recent study illustrated a mouse model of AATD with CRISPR editing of all five murine AAT genes [11], the antisense oligonucleotide (ASO) approach provides insights about short-term effects and dose dependent responses by targeting the common coding sequences. This ASO approach already demonstrated efficient reduction of human AAT transcript and successfully attenuated the liver injuries associated with AATD [12].

Although beneficial effects were previously found with AAT ASO treatment of liver disease, the knockdown of normal AAT may prove to be detrimental for the lung in the event of an injury with the overproduction of proteases, including chymotrypsin-like elastase 1 (*Cela1*), a digestive protease that is expressed during lung development [13]. *Cela1* was implicated with mouse lung development and human AATD [13, 21]. As the main etiology of COPD, smoking induces inflammation and tips the protease-antiprotease balance towards increased proteases activity. The underregulated protease activity leads to a distinct gene expression profile [14] and subsequent parenchymal destruction [15–17].

Another main trigger for rapid progression of COPD is respiratory viral or bacterial infection [18]. The viruses picornavirus and influenza are among the highest prevalence in patients with acute exacerbation of COPD [19]. Although both smoke exposure and additional influenza infection lead to increased protease production and result in parenchyma destruction and lung functional changes, the severity, cell types, and signaling pathways involved in initial inflammation and subsequent changes are quite distinct. Therefore, the impact of AAT ASO was assessed in both chronic smoke exposure as well as the smoke exposure with influenza injury models. Here we hypothesize that murine AAT ASO treatment decreases the expression of AAT and subsequently aggravates the smoke-induced lung injury and acute exacerbation modeled by influenza virus infection after smoke exposure.

With the mouse AAT ASO, we can understand if AAT knockdown significantly limits the availability of AAT in the lung. In addition, we can determine if AAT modulation affects the outcome of smoking-induced lung injury and acute COPD exacerbation in assessing the overall feasibility for using AAT ASO for treatment of liver disease in patients with a predisposition to proteolytic lung destruction.

## Materials and methods

### Alpha-1 antitrypsin antisense oligonucleotide treatment

Mouse AAT ASO and control ASO solution were prepared by Ionis Pharmaceutical in collaboration with Keith Blomenkamp and Dr. Jeff Teckman in Saint Louis University with the method described previously [12]. Two filter flasks were aliquoted upon arrival at 5 mg/ml concentration and administered via subcutaneous injection with a dose of 10 ml/kg body weight once a week, to make up a final concentration of 50 mg/kg.

### Animal housing and smoke exposure

**Fig 1:**
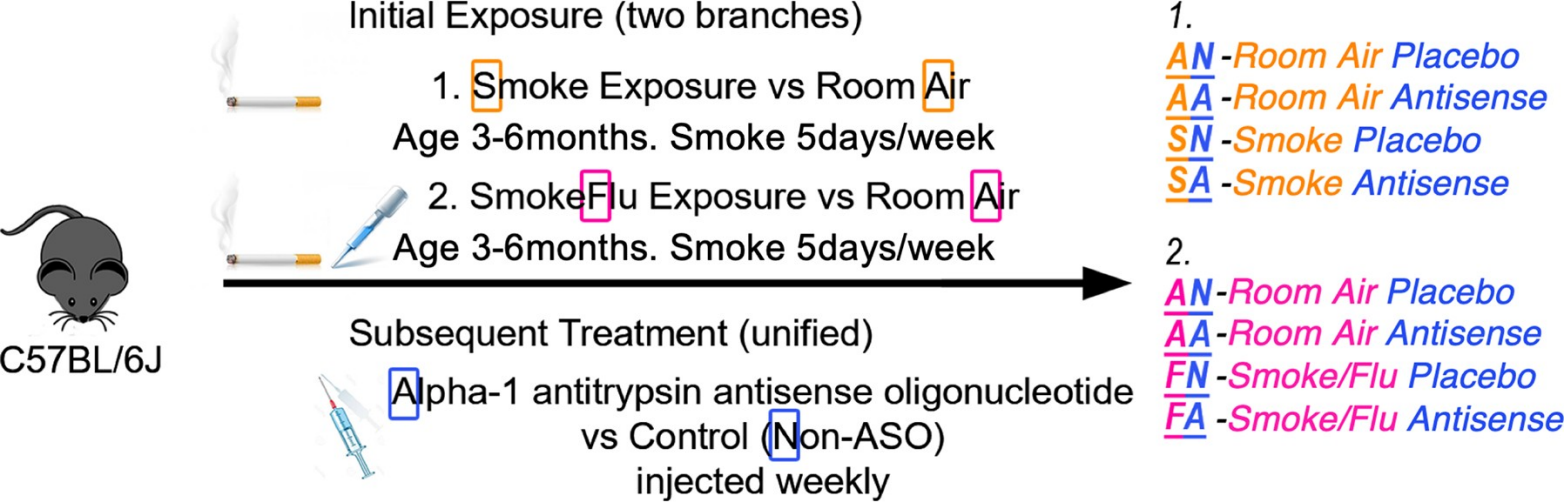
Experimental design. Alpha-1 antitrypsin antisense oligonucleotide treatment was tested in two lung injury models, smoke exposure for chronic obstructive pulmonary disease (COPD) and additional influenza infection for COPD exacerbation. Mice were randomly divided into 4 groups, first letter indicating the exposure or lung injury model (A for room air or S for smoke exposure; A for room air or F for smoke-flu exposure), second letter representing the treatment with or without ASO (A for AAT ASO or N for non-AAT control ASO).

All Animal studies were approved by and performed in compliance with guidelines from the Institutional Animal Care and Use Committee at Columbia University. 60 male C57BL/6J mice, 10–12 weeks of age, purchased from Jackson Laboratory (Bar Harbor, ME), were randomly divided into 4 groups, Air-NoASO (AN), Air-ASO (AA), Smoke-NoASO (SN), and Smoke-ASO (SA). All mice were acclimated to the animal facility for at least 48 hours prior to use and were given chow diet and water ad libitum. Smoke exposure was conducted on TE-10 Teague Smoking Apparatus (Teague Enterprises, Woodland, CA) with 3R4F Reference Cigarettes (University of Kentucky, Lexington, KY) for 5 days per week, 5 hours per day for a total of 3 months at a total particulate matter of 100~150 mg/m^3^. Total particulate matter was monitored by aerosol monitor DustTrak II 8530 (TSI, Shoreview, MN) and confirmed by gravimetric analysis. After starting the smoke exposure, alpha-1 antitrypsin antisense oligonucleotide treatment was administered weekly (Fig 1). This weekly dose was previously shown to dramatically reduce AAT in circulation [12].

### Influenza infection model

22 male C57BL/6J mice, 10–12 weeks of age, from Jackson Laboratory (Bar Harbor, ME), were randomly divided into 4 groups for another set of comparisons, Air-NoASO (AN), Air-ASO (AA), SmokeFlu-NoASO (FN), and SmokeFlu-ASO (FA). After smoke exposure for two weeks as described above, mice were anesthetized with isoflurane (1–5%) and infected intranasally with a dose of 1000 TCID50 Influenza A/Puerto Rico/8/1934 H1N1 virus suspension in 30μl of PBS. All laboratory personnel who perform infections and handle infected mice have undergone training for BSL-2 procedures to enter and use animals in this facility. Two oligonucleotide doses were given 1 and 4 days prior to influenza infection and continued once a week post-infection to reach the pre-defined endpoint of day 18 (Fig 1). As the infection dose may still result in animal death, starting from the first antisense treatment which is 4 days before the infection, animals were weighed and monitored for health on a daily basis by qualitative assessment of general health noting clinical signs of morbidity such as ruffled fur, hunched posture and shivering. The humane endpoint was defined as 20% weight loss and moribund appearance as assessed by lack of movement, hunched posture, and unkempt appearance. If within 24 hours mice did not recover and regain weight, they were euthanized.

### Lung function testing

We assessed the lung function of 60 mice for smoke-antisense study. After 3 months of smoke exposure and weekly injections, lung function was measured with flexiVent FX2 (EMKA, Montréal, QC, Canada), a computer-controlled piston ventilator that measures lung function in small animals. The mice were sedated with pentobarbital 75 mg/kg, tracheostomized and intubated with an 18-gauge beveled tracheal tube to connect directly to flexiVent. After 3–5 min equilibration on the ventilator with a tidal volume of 8 ml/kg and frequency of 150 breaths/min and maintaining the mice at 37°C with a homeothermic blanket (Homeothermic Blanket System; Harvard Apparatus, Holliston, MA), mice were given succinylcholine 0.5 mg every 14 min by i.p. injection. Perturbations, including deep inflation, snapshot-150, quick prime-3, and PV loop (PVs-P) were performed using the flexiVent system. Measurements were repeated until we obtained 3 consecutive consistent and valid (COD>0.95) readings for each perturbation.

### Inflammation and emphysema assessment of the lung

For both smoke-antisense and smoke-flu-antisense studies, lungs were lavaged during sacrifice once with 500μl of PBS to obtain concentrated BAL supernatant, followed by two more lavages with 1 ml of sterile PBS to maximize cell yields. Total cells were counted with a TC20 automatic cell counter (Bio-Rad, Philadelphia, PA). Cytospin preparations were stained with Quick-Diff (Imeb), and cells were analyzed for differential counts using morphological criteria. Left lung was formalin-fixed, paraffin-embedded, step-sectioned every 200μm and stained by H&E method. Morphometric analyses, including the traditional gridline counting and parenchymal airspace profiling methods [20], were performed on the lung sections. We used whole slide scans in which each scan is equivalent to 20–100 fields of view under a microscope of the same magnification. With 13–15 biological replicates per group to examine, we randomly sampled two sections at different depths from each sample block. Parenchymal airspace profiling is a novel morphometric method that categorizes airspaces by size with machine learning algorithms into subpopulations of single alveolus, alveolar sac, and ductal/destructive airspaces [20]. Additional parameters, including representative size and area fraction, for each categories of airspace are calculated accordingly.

### Western blotting, ELISA, protease activity assay, and qRT-PCR

The AAT protein expression in the lung was assessed with western blotting (alpha-1 antitrypsin antibody, #16382-1-AP, rabbit polyclonal, Proteintech, Rosemont, IL). Serum level of AAT was evaluated with Mouse Alpha-1 Antitrypsin ELISA Kit (ab205088, Lot Number: GR3272327, Abcam, Cambridge, United Kingdom). Elastase activity assays were performed per the manufacturer’s instructions using EnzChek Elastase Assay Kit (E-12056, ThermoFisher Invitrogen, Waltham, MA) from whole lung homogenates. PCR probe for *Cela1* (chymotrypsin-like elastase family, member 1) was purchased from ThermoFisher (Assay ID: Mm00712898_m1, Cat #4331182, Applied Biosystems, Foster City, CA).

### Statistical analysis

Two-way ANOVA (exposure*treatment) followed by Tukey post hoc tests were performed to test for significant differences (alpha < 0.05) between exposure and treatment groups. A two-tailed student’s t-test with Bonferonni adjusted alpha was utilized for comparing experimental groups to the reference group in real-time PCR experiments. GraphPad Prism 8 was utilized to perform the log-rank test (Mantel-Cox) for survival curve analysis. All error bars indicate mean ± SEM.

## Results

### ASO treatment effectively knocked down AAT protein expression, and increased both Cela1 expression and elastase activity in the lung

**Fig 2:**
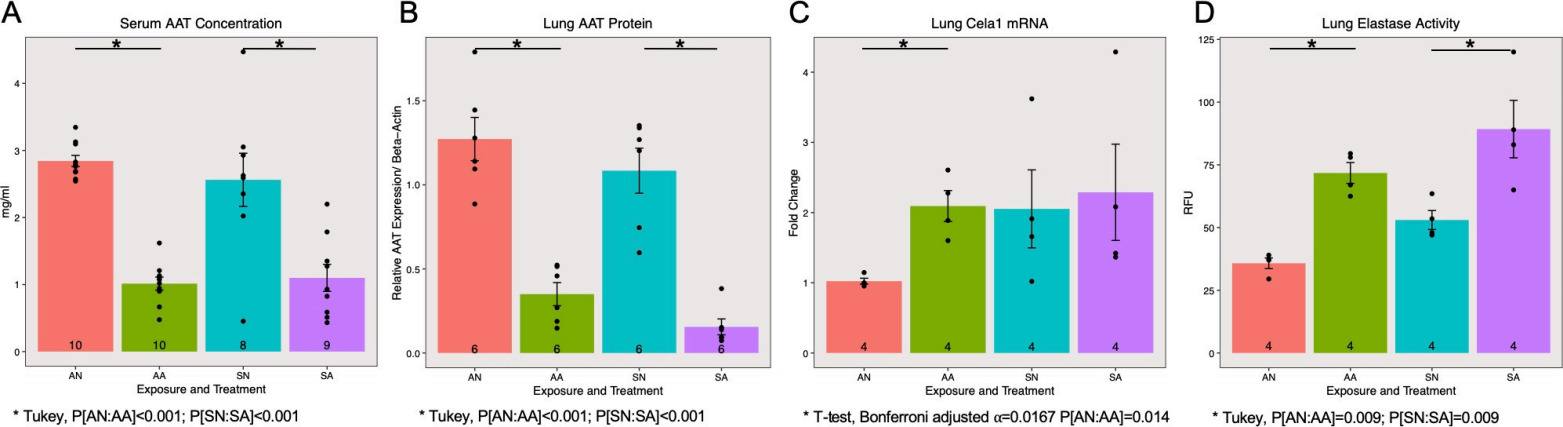
AAT ASO knocks down AAT in smoke-induced COPD model. Alpha-1 antitrypsin (AAT) expression was significantly decreased by the antisense oligonucleotide treatment in both serum (A, P[treatment]<0.001) and lung (B, P[treatment]<0.001) samples. The mRNA level of chymotrypsin-like elastase 1 (*Cela1*) was significantly increased by AAT antisense from baseline controls (C, P[ANvsAA] = 0.014) and elastase activity in the lung was significantly increased by both smoke exposure (D, P[exposure] = 0.020) and antisense treatment (D, P[treatment]<0.001).

Endogenous AAT protein expression in the serum (Fig 2A ANOVA, P[treatment]<0.001, N = 8–10 per group) and the lung (Fig 2B ANOVA, P[treatment]<0.001; N = 6 per group, from 3 western blots; S2 Fig) were significantly reduced in mice injected with ASO. Tukey’s HSD post hoc tests showed a significant reduction in AAT levels among air exposed and smoke exposed mice treated with ASO compared to control treated animals with matching exposures (P<0.001 in each case). ASO injections also significantly increased the *Cela1* expression in the lungs of the air-exposed mice, but not for the smoke-exposed mice (Fig 2C student’s t-test P[Room Air-Placebo: Room Air-Antisense] = 0.014, N = 4 per group). Elastase activity was significantly increased by smoke exposure (Fig 2D ANOVA, P[exposure] = 0.020) and ASO treatment (ANOVA, P[treatment]<0.001;). Tukey’s HSD post hoc tests showed a significant increase in elastase activity among air exposed (P[Room Air-Placebo: Room Air-Antisense] = 0.009) and smoke exposed (P[Smoke-Placebo: Smoke-Antisense] = 0.009) mice treated with ASO compared to control treated animals with matching exposures.

### ASO did not exacerbate smoke-induced lung injury

**Fig 3:**
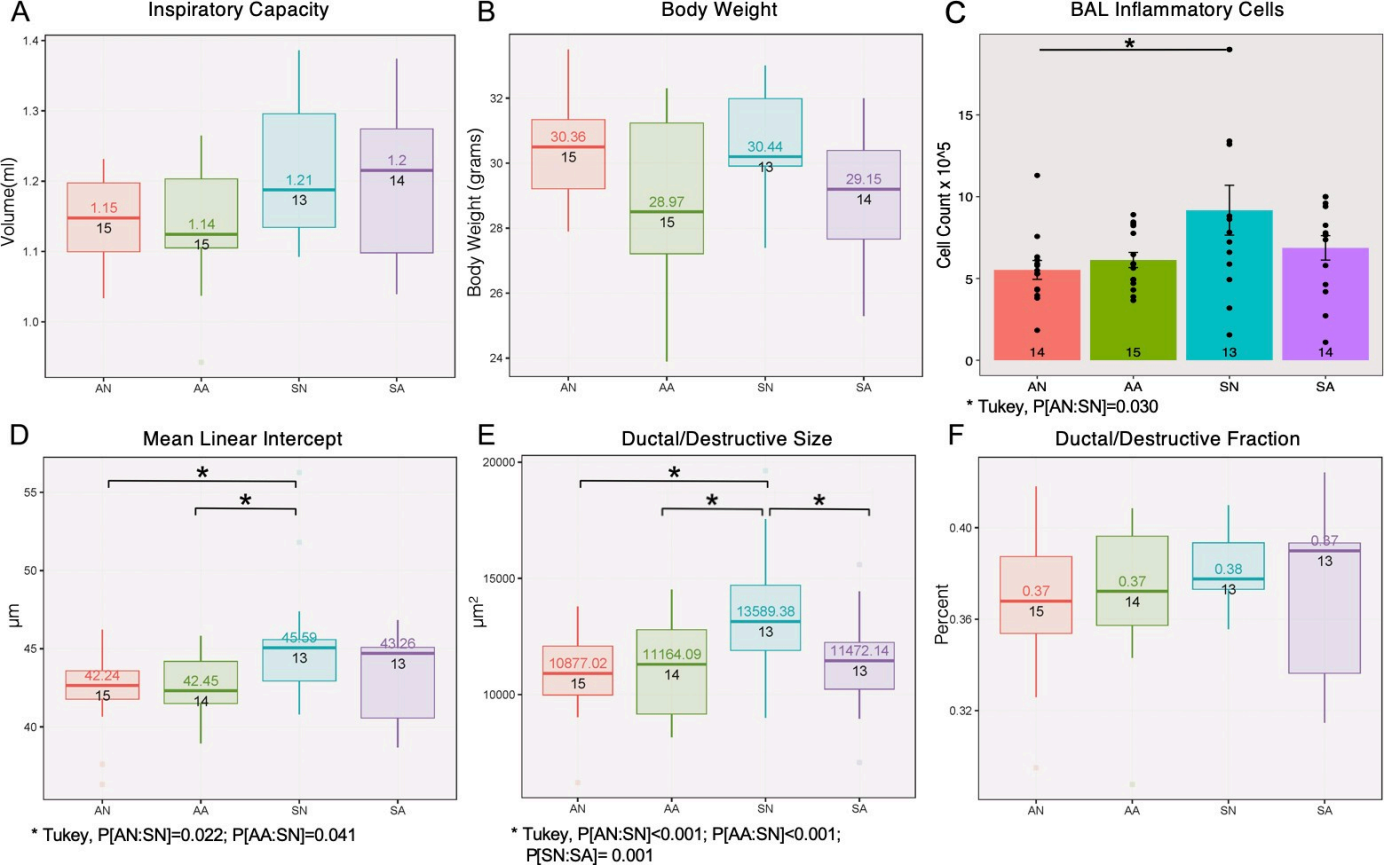
Effects of AAT ASO knockdown in smoke-induced COPD model. Smoke exposure contributes to changes in inspiratory capacity (A, volume, P[exposure] = 0.017), inflammation (C, bronchoalveolar lavage cell counts, P[exposure] = 0.020) and emphysema (D-E, mean linear intercept, P[exposure] = 0.011; ductal/destructive size, P[exposure]<0.001). Antisense treatment contributes to changes in body weight (B, P[treatment] = 0.015) and ductal/destructive size (E, P[treatment] = 0.015).

Lung function testing results measured by flexiVent showed an overall significantly increased inspiratory capacity (Fig 3A ANOVA, P[exposure] = 0.017, N = 13–15 per group) for mice exposed to smoke. ASO treatment did not contribute to any significant changes in inspiratory capacity or resistance regardless of the exposure conditions.

Inflammatory cell counts in the bronchoalveolar lavage fluid (BALF) were only significantly increased among antisense mice exposed to smoke compared to those exposed to room air (Fig 3C ANOVA, P[exposure] = 0.019; Tukey’s HSD P[Room Air-Placebo: Smoke-Placebo] = 0.03). Analysis of the lung morphometry in the mice demonstrated emphysema development with smoke exposure alone for Mean Linear Intercept (Fig 3D ANOVA, P[exposure] = 0.011; Tukey’s HSD P[Smoke-Placebo: Room Air-Antisense] = 0.041 and P[Smoke-Placebo: Room Air-Placebo] = 0.022) and with both exposure & treatment factors for Ductal/destructive Size (Fig 3E ANOVA, P[exposure]<0.001, P[treatment] = 0.015, P[treatment*exposure]<0.004; Tukey HSD P[Smoke-Placebo: Room Air-Antisense]<0.001, P[Smoke-Placebo: Smoke-Antisense] = 0.001, and P[Smoke-Placebo: Room Air-Placebo]<0.001). Although the ASO treatment successfully decreased the expression of AAT in the serum and the lung, no additional exacerbation of lung injury was observed as a result of treatment with ASO treatment with smoke exposure (Fig 3A–3F).

### ASO did not significantly affect survival and lung responses of mice exposed to smoke and influenza infection

**Fig 4:**
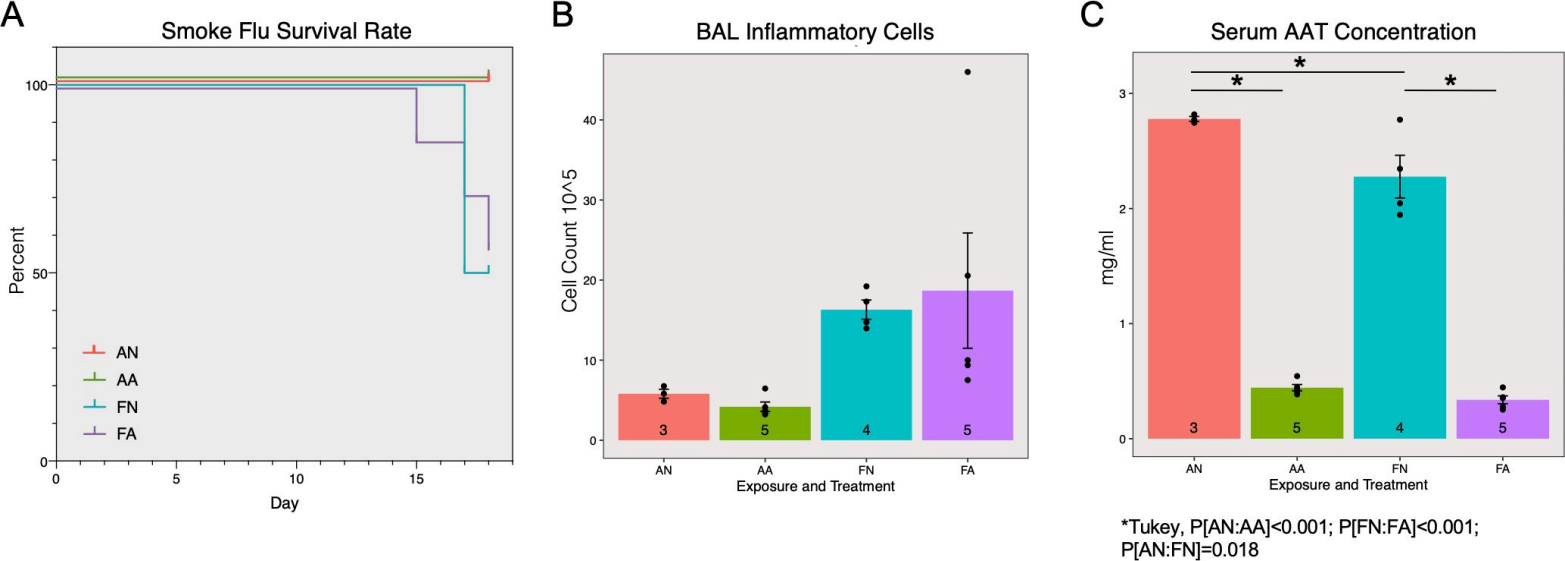
Effects of AAT ASO on smoke and influenza induced COPD exacerbation. The survival rate (A) was only significantly affected by smoke-flu injury but not antisense treatment. Inflammatory cell counts (B) were significantly affected by smoke-flu injury (P[exposure] = 0.025), but not antisense treatment (P[treatment] = 0.810). Serum AAT concentrations were significantly decreased by the antisense oligonucleotide treatment (C, P[treatment]<0.001).

Two weeks after the infection, 4 out of 8 SmokeFlu-NoASO (FN) mice and 3 out of 8 SmokeFlu-ASO (FA) mice died abruptly before reaching pre-defined humane endpoint. The control groups, 3 Air-NoASO (AN) and 5 Air-ASO (AA) did not have adverse effects. Both groups exposed to smoke and influenza virus exhibited mortality of ~50% two weeks after the infection and had no significant difference in survival rate (Fig 4A Log-Rank p = 0.1754).

Similar to smoke exposure alone, there was significantly increased inflammation due to inhaled exposure to smoke and flu (Fig 4B ANOVA, P[exposure] = 0.025). However, the ASO treatment did not contribute to any significant changes (ANOVA, P[treatment] = 0.810). A significant reduction in serum AAT concentration was also observed as a result of ASO treatment (Fig 4C ANOVA, P[treatment]<0.001, Tukey’s HSD P[Room Air-Placebo: Room Air-Antisense]<0.001, P[Smoke Flu-Placebo: Smoke Flu-Antisense]<0.001, and P[SmokeFlu-Placebo: Room Air-Placebo]<0.018).

## Discussion

Consistent with the previous study [12], where an 80% reduction in circulating AAT was found in the liver after AAT ASO treatment, we also found more than 50% reduction in AAT protein expression in the serum and the lung (Fig 2A and 2B). As one of the digestive enzymes that can be covalently neutralized by AAT and important in emphysema development, we first examined the expression of chymotrypsin-like elastase 1 (*Cela1*) [21]. ASO treatment significantly up-regulated *Cela1* expression in room air-exposed mice to a comparable level to those exposed to smoke. The treatment did not significantly change *Cela1* expression levels among smoke exposed mice, suggesting partial knockdown of the protease inhibitor does favor further over-expression of *Cela1* (Fig 2C). From another perspective, the AAT expression is still present at a detectable level (Fig 2A and 2B). This level of AAT may remain effective and functional, especially in comparison to a recently published paper showing complete knock-out of AAT resulted in significant changes in lung function and emphysema [11].

In the mouse smoke exposure model of COPD, we did not observe any significant differences in lung functional parameters, inflammatory cells, or lung structural changes attributable to ASO treatment. As expected the differences between smoke alone and air control can be found in degrees of inflammation and emphysema. Partial knockdown of AAT only significantly modulated ductal/destructive size among smoke exposed mice, but did not otherwise affect the inflammatory and emphysematous metrics measured in the smoke exposure model (Fig 3). In order to examine whether partial knockdown of AAT has an effect during acute COPD exacerbation we designed another set of experiments that utilize both smoke and influenza virus exposure. The exacerbation model itself clearly distinguished the mice exposed to smoke and influenza from room air controls even with limited survival rate (~50%). However, the ASO treatment did not have a statistically significant effect for mice exposed to both smoke and flu. A lower than anticipated survival rate in the smoke and flu exposed animals reduced the sample size. Subsequent studies should account for reduced survival when performing their power analysis. Further, future studies may also benefit from powering their experiments to allow stratification by gender when exploring sex specific effects of ASO on the lung [22].

This study was designed to identify the potentially harmful effects of AAT knockdown on the lung in the presence of acute exacerbation or chronic COPD. A potential explanation for the limited effect of ASO treatment on the lung may be a result of off-target binding and compensatory mechanisms in response to the treatment. The antisense design may target unintended proteins with similar nucleotide sequence. These potential mechanisms need to be investigated in order to fully understand the protease-antiprotease imbalance pertaining to AAT in the murine smoke exposure model [23].

In summary, ASO presents an effective method for partial knockdown of AAT expression and a valuable vehicle for mechanistic studies regarding AAT mediated protease-antiprotease balance. In both scenarios that we investigated, AAT modulation did not significantly alter the measured outcomes of smoke-induced lung injury and additional viral infection. Further, significantly reduced AAT expression was associated with increased neutrophil elastase activity and may impact the expression of other key proteases. Considering the *Cela1* expression and lung elastase activity results, it is possible that the partial knockdown of AAT by ASO was not sufficient to promote significant worsening of lung injury during the timeframe observed. However, the observation that ASO knockdown of AAT led to an increase in elastase activity suggests that extended treatments might eventually lead to structural changes or damage in the lung.

Most importantly, this study did not demonstrate significant worsening of lung injury in murine models of COPD exacerbations as a result of reduced AAT expression. These results may support the idea that partial knockdown of AAT expression in PiZZ patients for liver protection may not have detrimental effects on the lung. Therefore, the use of AAT ASO treatment in models of AAT Z-polymer associated liver disease warrants further exploration to determine if there is a benefit of this therapy in combination with AAT augmentation therapy for individuals at risk for COPD development.

## Supporting information

S1 Fig AAT ASO treatment decreased AAT expression in the lungs of smoke exposed mice. Western blots contributing to Fig 2B. Western blot detecting AAT and ß Actin in mouse lung homogenates.
(PDF)

S2 Fig No significant differences in lung function in AAT ASO treated and smoke exposed mice. FlexiVent measurements were repeated at least 3 times, in order to obtain 3 consecutive consistent and valid (COD>0.95) readings. No significant differences as a result of exposure or treatment were observed. A) Quasi-Static Compliance. B) Newtonian Resistance. C) Resistance. D) Tissue Damping. E) Compliance. F) Tissue Elastance.
(PDF)

S3 Fig Decreased AAT expression in the lungs of mice treated with AAT ASO and exposed to smoke and flu. A) Western blot detecting AAT (upper) and ß Actin (lower) in mouse lung homogenates. B) Densitometry performed on the western blot.
(PDF)

S1 Data (XLSX)

S1 Raw images (PDF)
