# Supplementary figures and images for: Knockdown of Alpha-1 Antitrypsin with antisense oligonucleotide does not exacerbate smoke induced lung injury
Source: PLoS One. 2021 Feb 4;16(2):e0246040. doi: 10.1371/journal.pone.0246040 (PMC7861354; doi:10.1371/journal.pone.0246040)

S1 Figure

AAT and Beta Actin

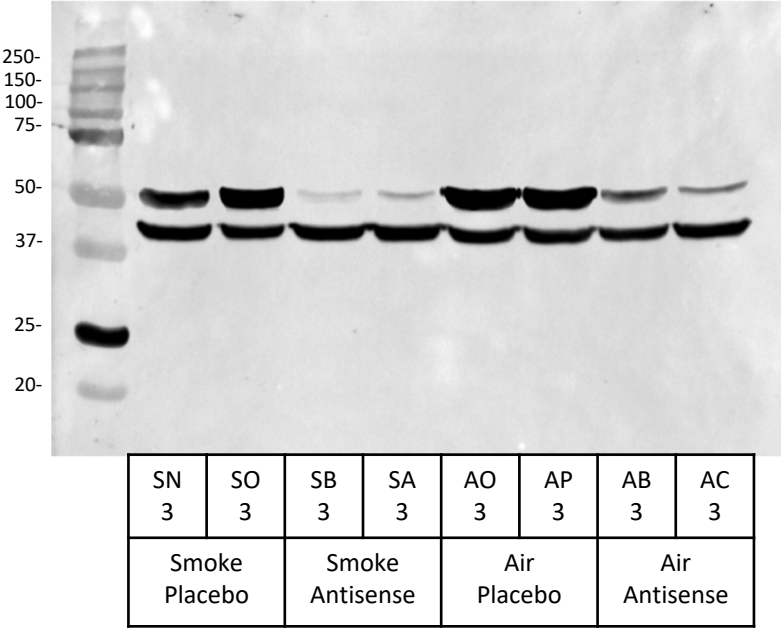

AAT

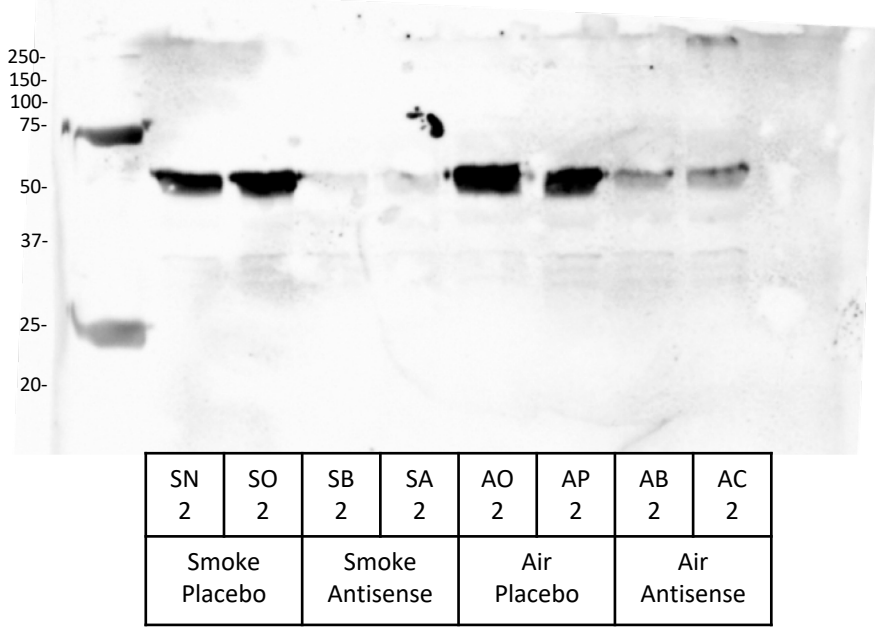

AAT

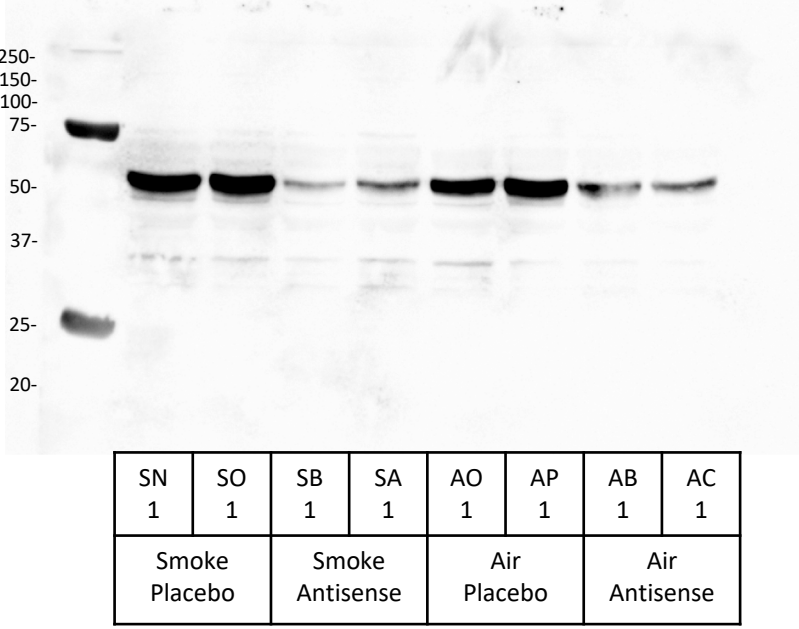

Beta Actin

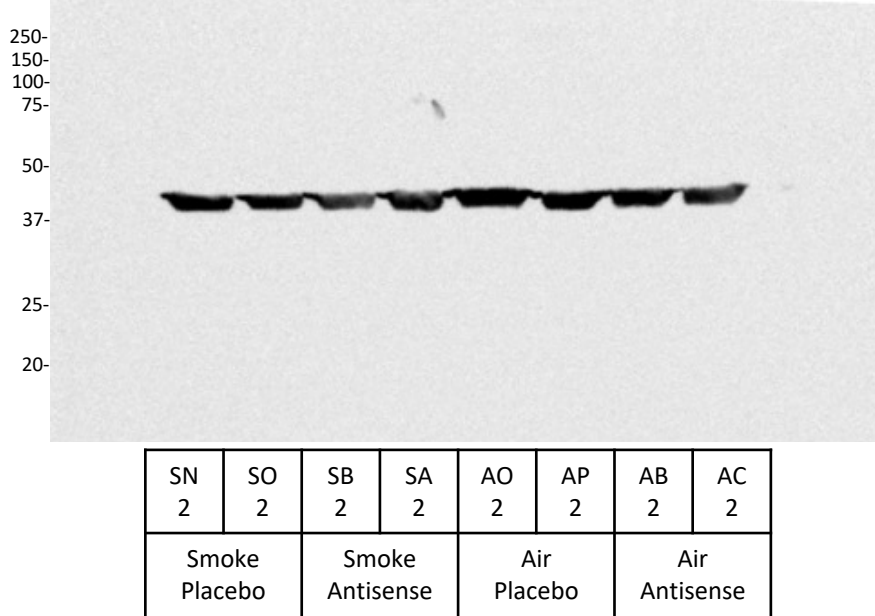

Beta Actin

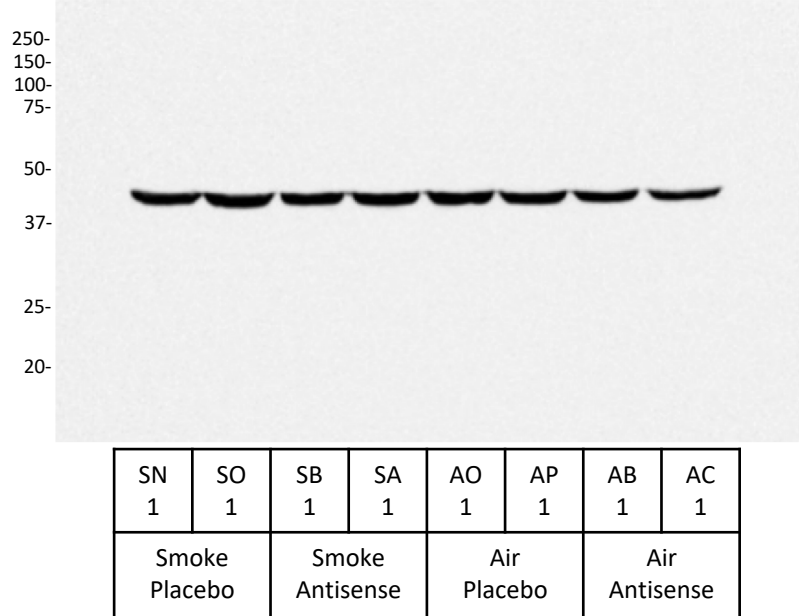

Supplement: S1 Fig — Western blots contributing to Fig 2B. Western blot detecting AAT and ß Actin in mouse lung homogenates. (PDF) [file pone.0246040.s001.pdf]

S2 Figure

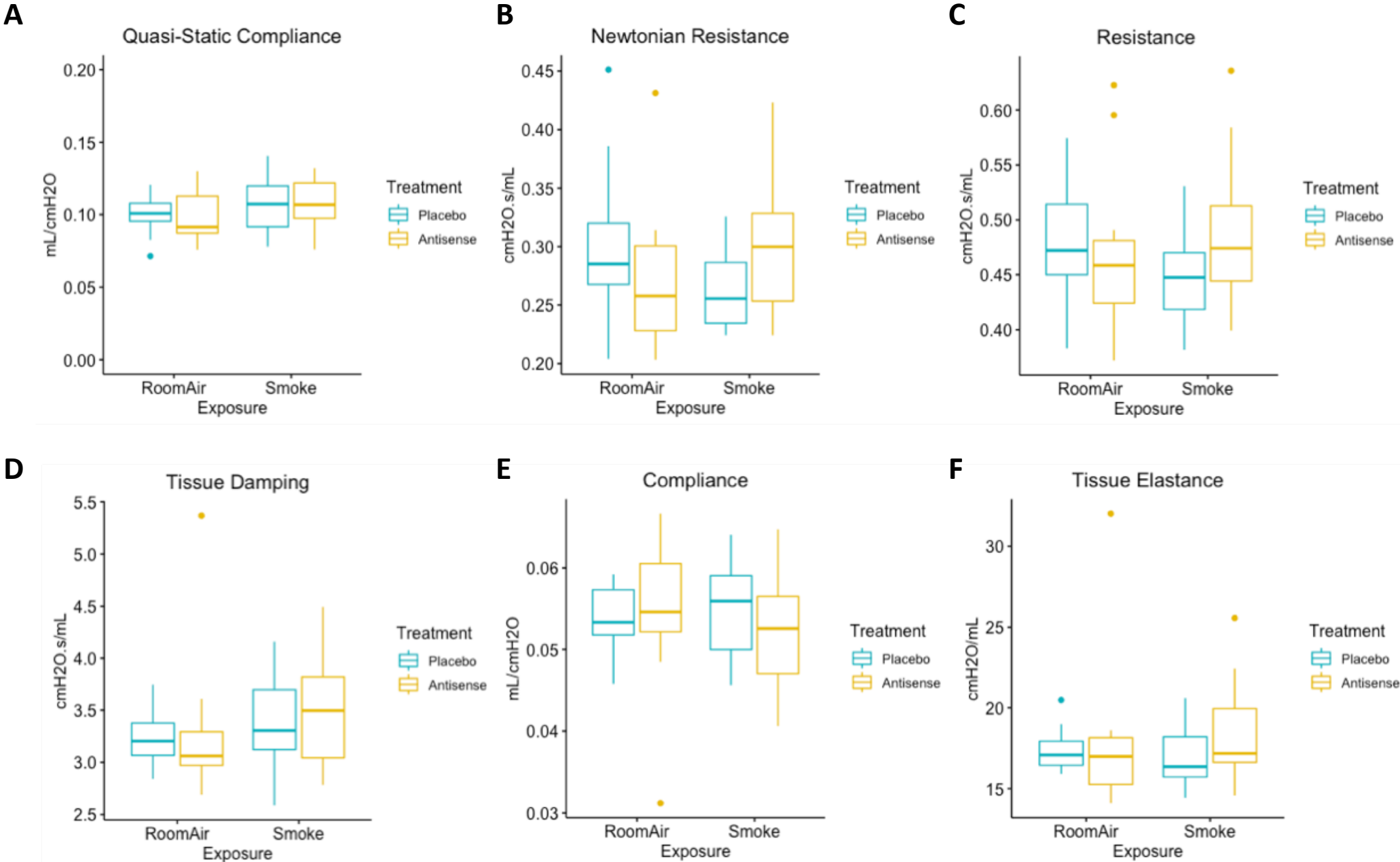

Supplement: S2 Fig — FlexiVent measurements were repeated at least 3 times, in order to obtain 3 consecutive consistent and valid (COD>0.95) readings. No significant differences as a result of exposure or treatment were observed. A) Quasi-Static Compliance. B) Newtonian Resistance. C) Resistance. D) Tissue Damping. E) Compliance. F) Tissue Elastance. (PDF) [file pone.0246040.s002.pdf]

S3 Figure

A

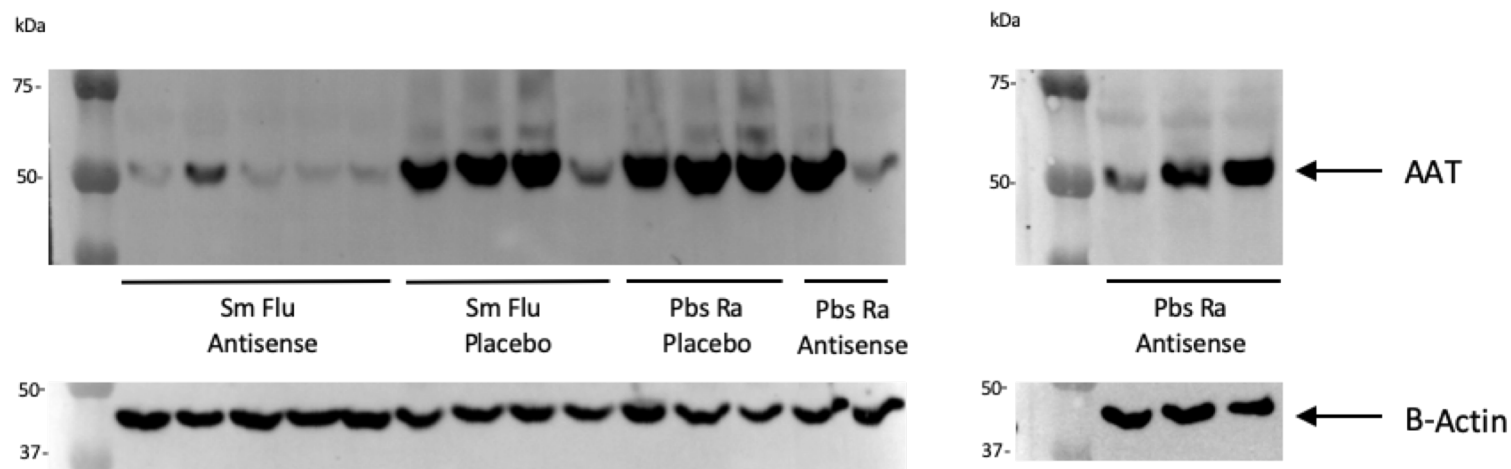

B

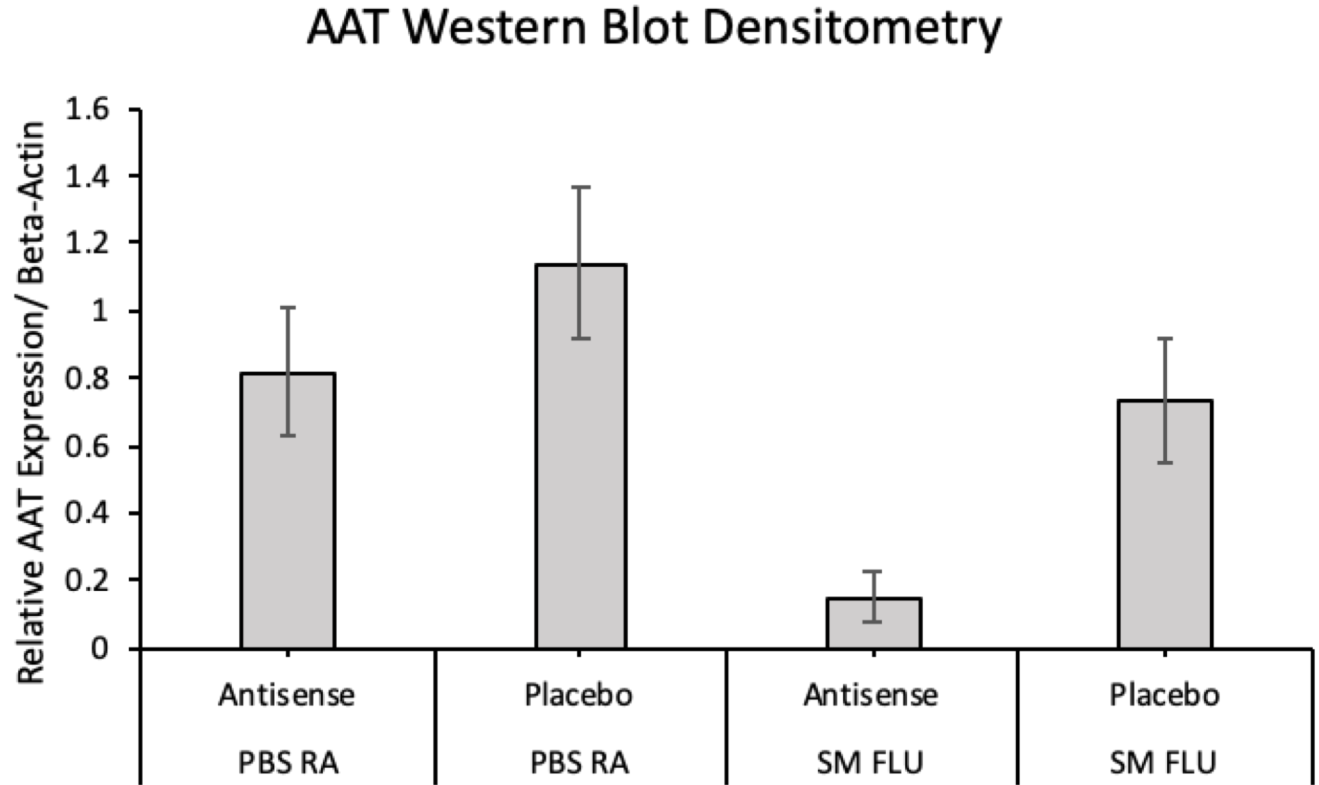

Supplement: S3 Fig — A) Western blot detecting AAT (upper) and ß Actin (lower) in mouse lung homogenates. B) Densitometry performed on the western blot. (PDF) [file pone.0246040.s003.pdf]
