# Supplementary material for: Knockdown of Alpha-1 Antitrypsin with antisense oligonucleotide does not exacerbate smoke induced lung injury
Source: PLoS One. 2021 Feb 4;16(2):e0246040. doi: 10.1371/journal.pone.0246040 (PMC7861354; doi:10.1371/journal.pone.0246040)
Supplement: S1 Raw images — (PDF) [file pone.0246040.s005.pdf]

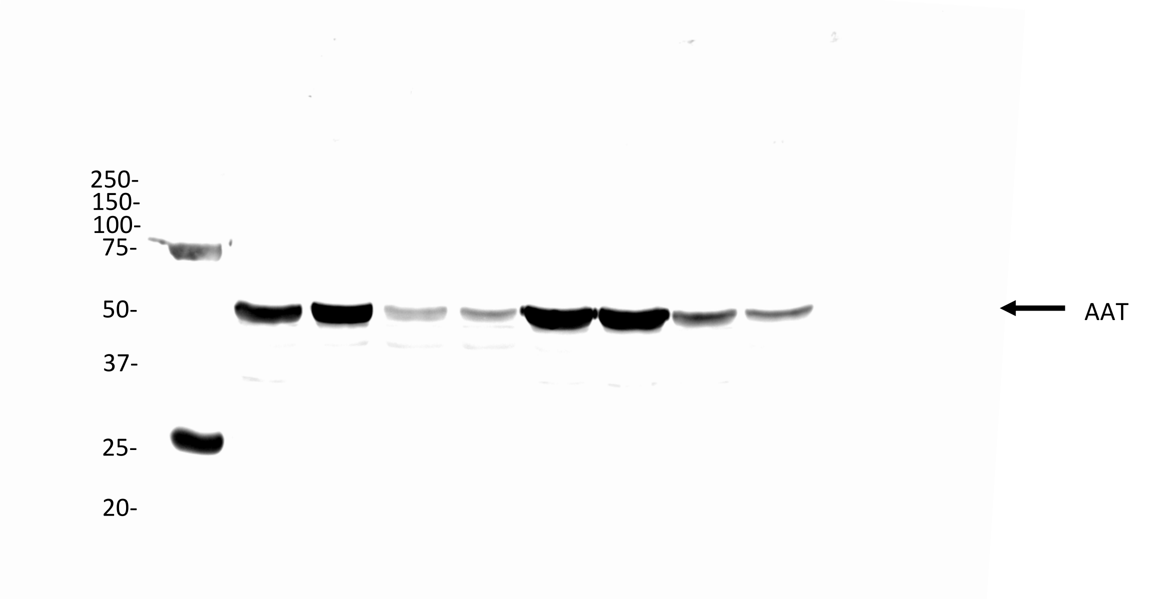

SN 3	SO 3	SB 3	SA 3	AO 3	AP 3	AB 3	AC 3
Smoke Placebo		Smoke Antisense		Air Placebo		Air Antisense	

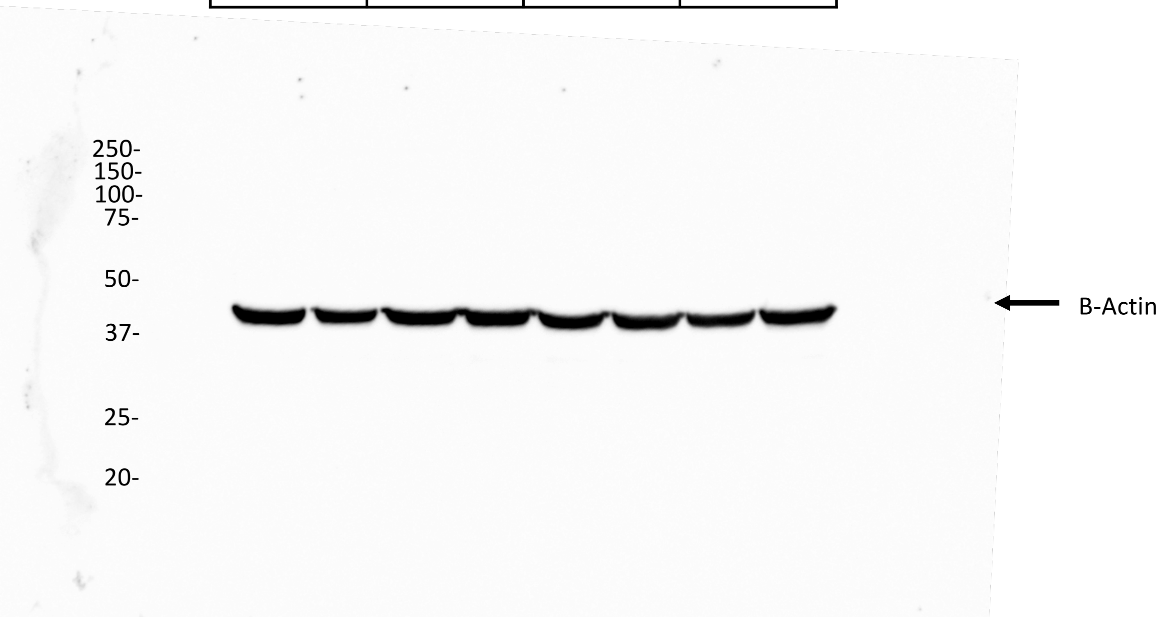

SN 3	SO 3	SB 3	SA 3	AO 3	AP 3	AB 3	AC 3
Smoke Placebo		Smoke Antisense		Air Placebo		Air Antisense	

Raw Western Blot Images Used to Generate Figure 2B
Images captured with GE Healthcare
ImageQuant LAS 4000
Chemiluminescence
Exposure Type: Increment

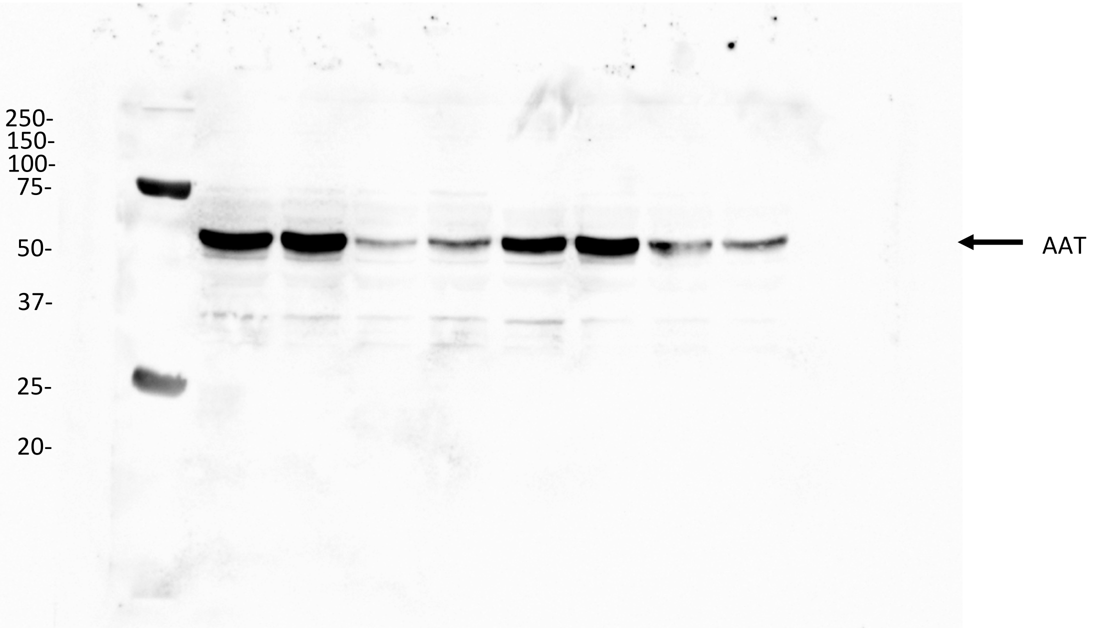

SN 1	SO 1	SB 1	SA 1	AO 1	AP 1	AB 1	AC 1
Smoke Placebo		Smoke Antisense		Air Placebo		Air Antisense	

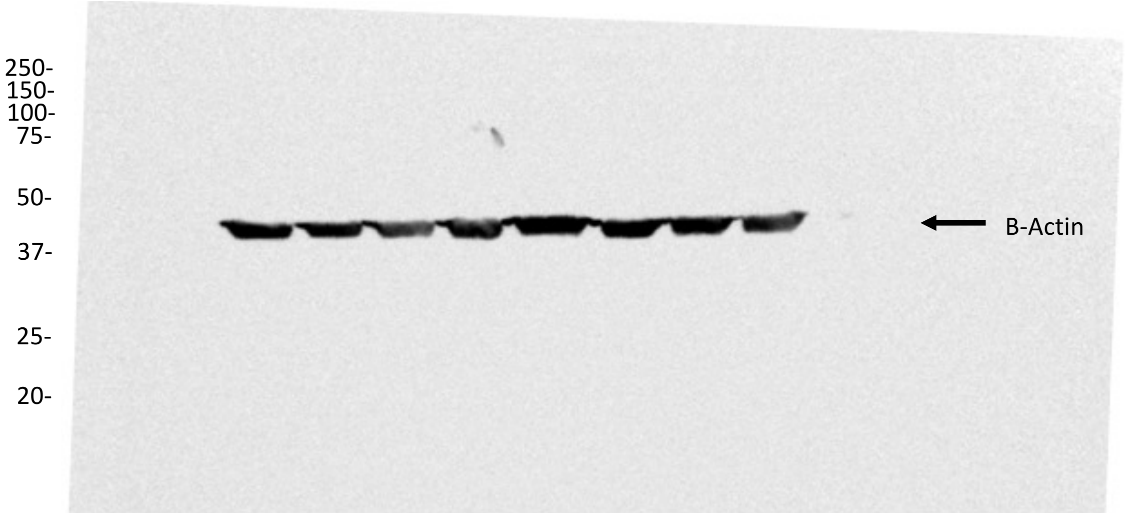

SN 2	SO 2	SB 2	SA 2	AO 2	AP 2	AB 2	AC 2
Smoke Placebo		Smoke Antisense		Air Placebo		Air Antisense	

Raw Western Blot Images Used to Generate Figure 2B
Images captured with GE Healthcare
ImageQuant LAS 4000
Chemiluminescence
Exposure Type: Increment

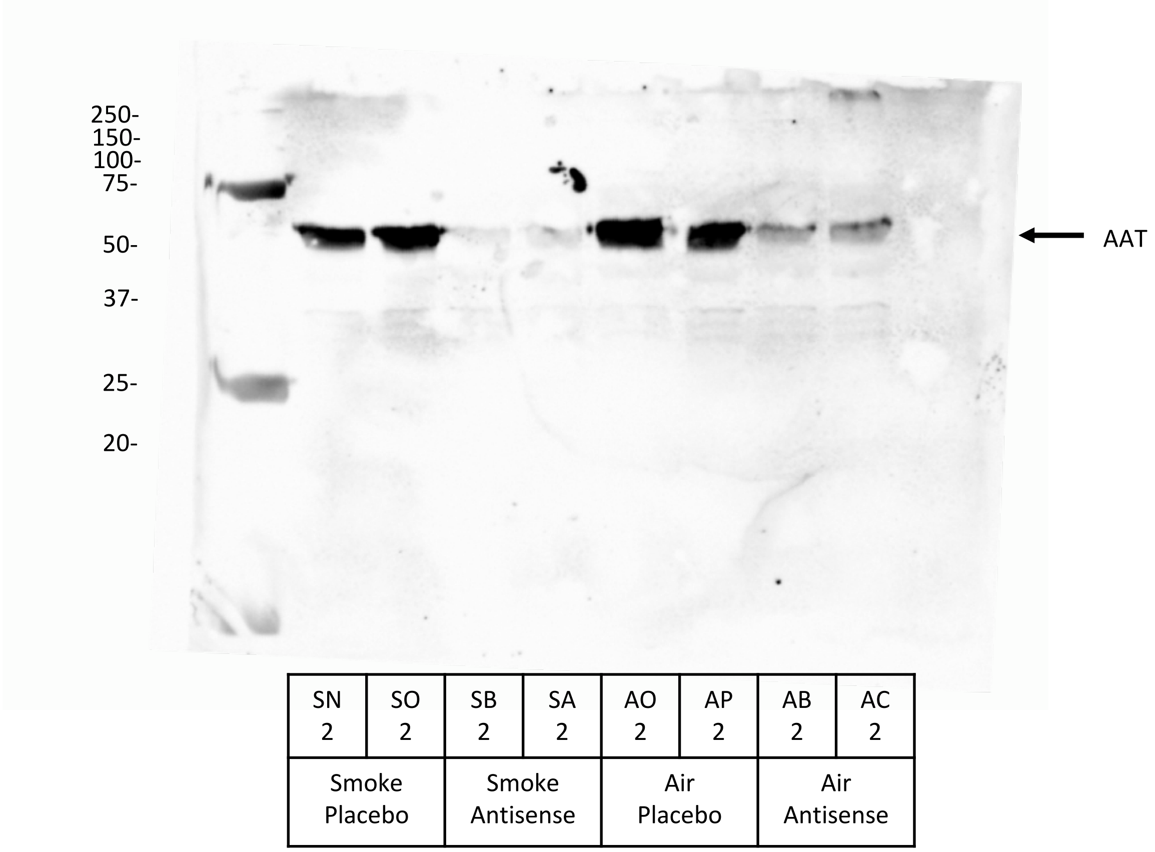

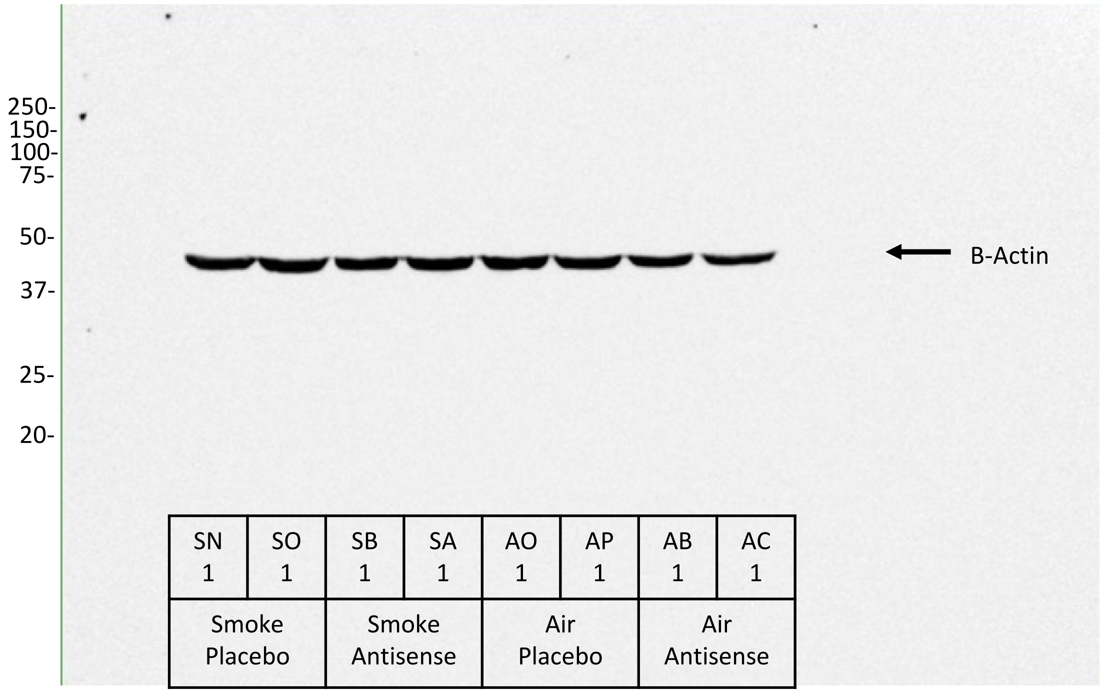

Raw Western Blot Images Used to Generate Figure 2B
Images captured with GE Healthcare
ImageQuant LAS 4000
Chemiluminescence
Exposure Type: Increment

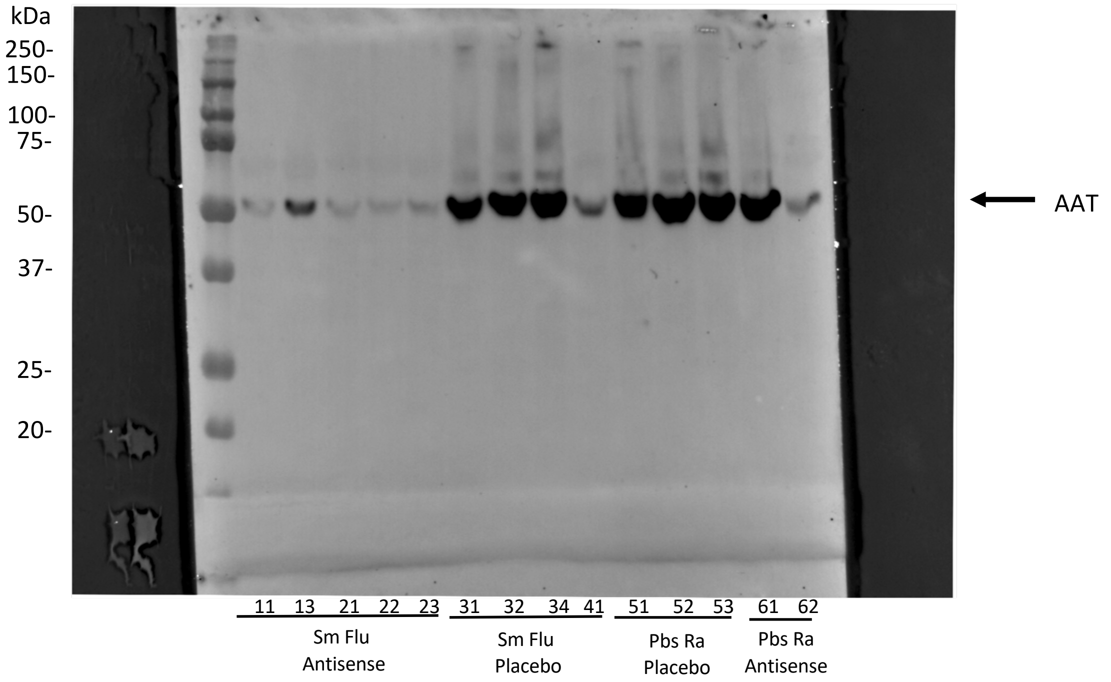

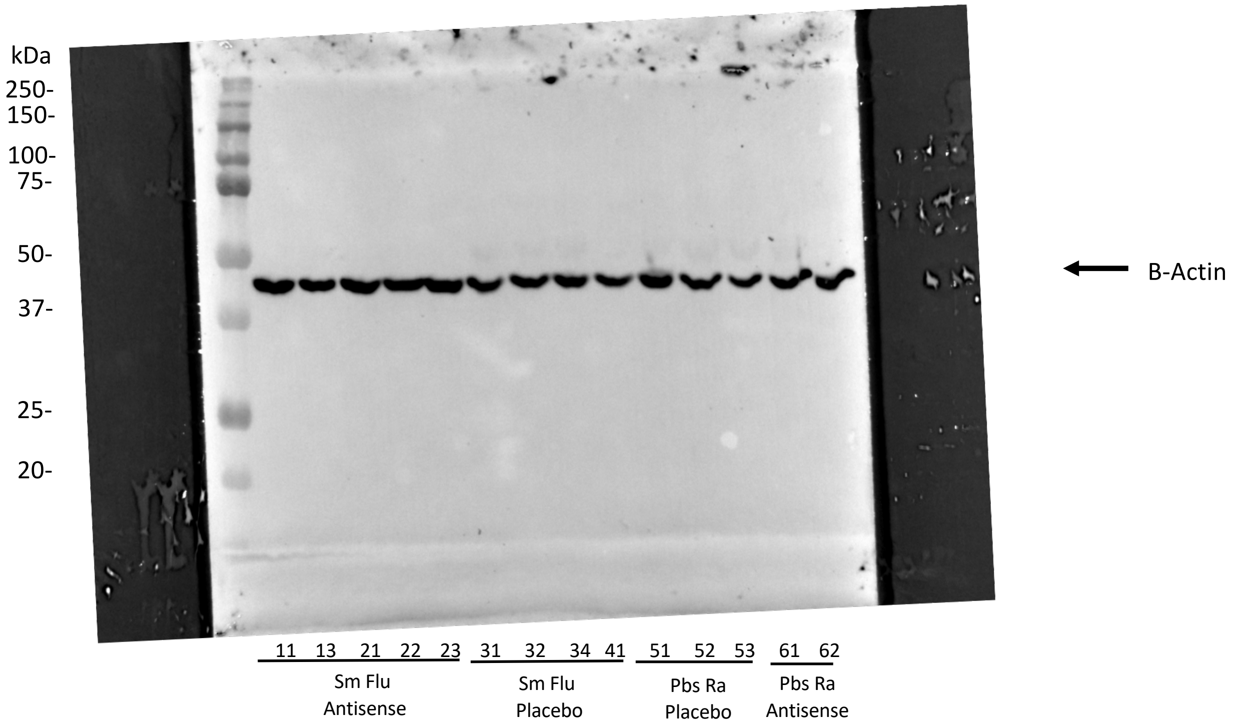

Raw Western Blot Images Used to Generate Figure S3
Images captured with GE Healthcare
ImageQuant LAS 4000
Chemiluminescence
Exposure Type: Increment

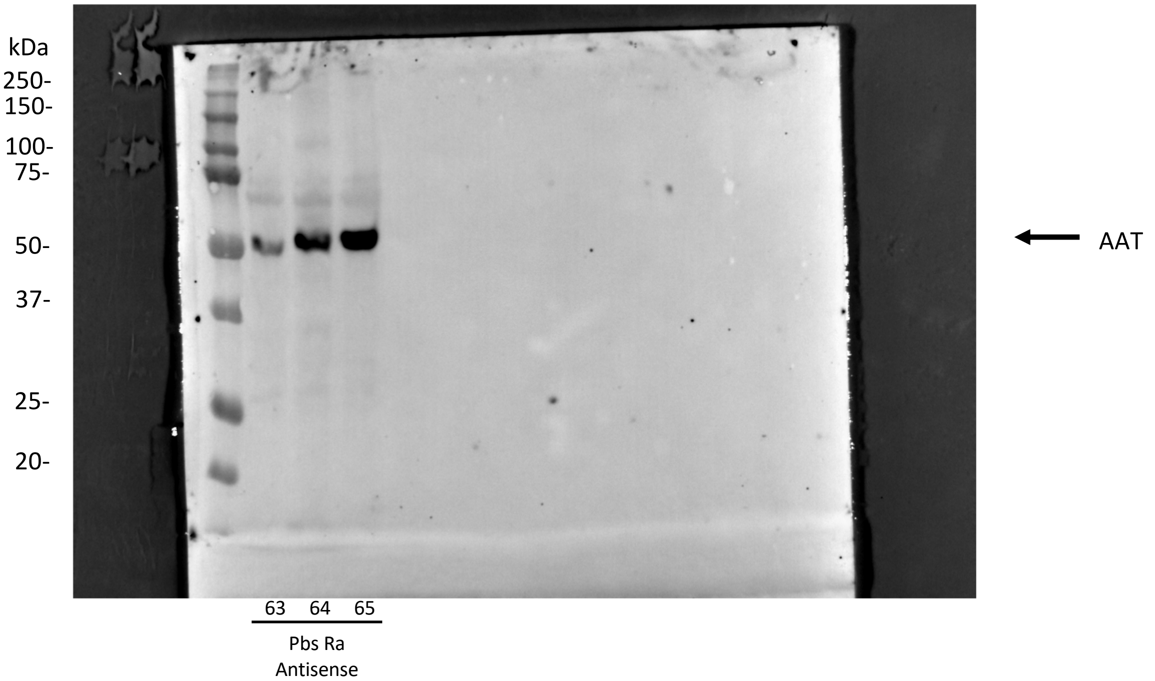

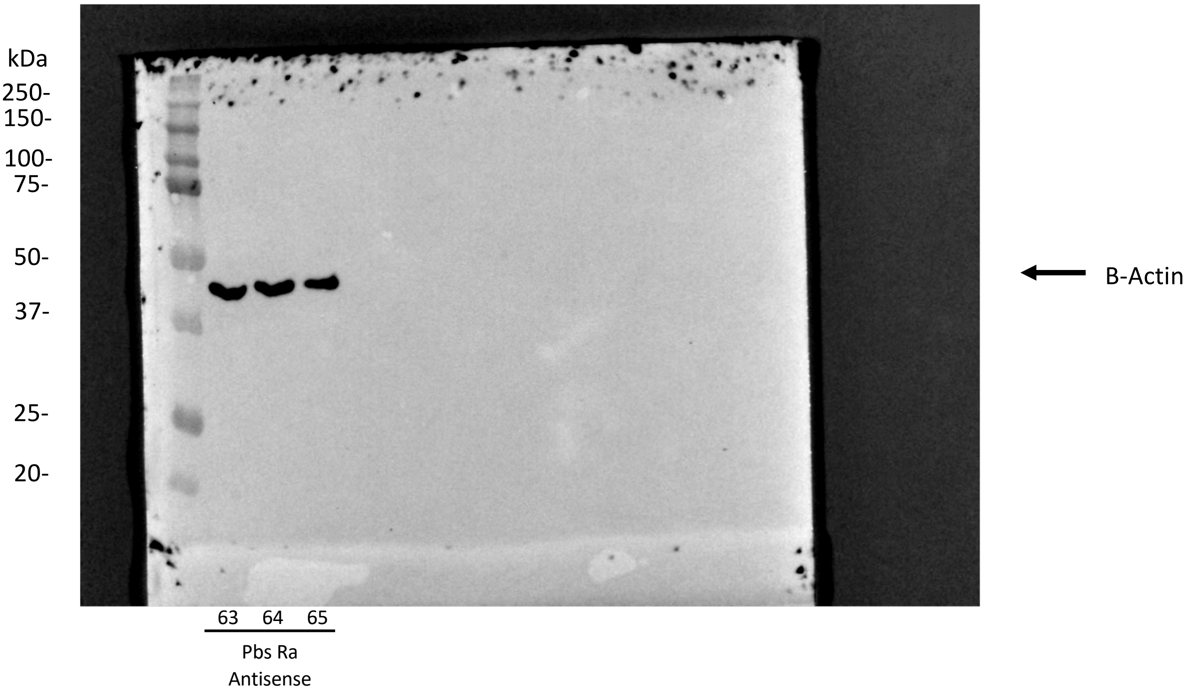

Raw Western Blot Images Used to Generate Figure S3
Images captured with GE Healthcare
ImageQuant LAS 4000
Chemiluminescence
Exposure Type: Increment
